# The Impact of the COVID-19 Pandemic on Oldest-Old Social Capital and Health and the Role of Digital Inequalities: Longitudinal Cohort Study

**DOI:** 10.2196/62824

**Published:** 2025-01-09

**Authors:** Luca Guido Valla, Michele Rossi, Alessandra Gaia, Antonio Guaita, Elena Rolandi

**Affiliations:** 1 Department of Statistical Sciences University of Padua Padua Italy; 2 Golgi Cenci Foundation Abbiategrasso Italy; 3 Centre for Longitudinal Studies UCL Social Research Institute University College London London United Kingdom; 4 Department of Sociology and Social Research University of Milano-Bicocca Milan Italy; 5 Department of Brain and Behavioral Sciences University of Pavia Pavia Italy

**Keywords:** older adults, information and communication technology, ICT, ICT use, COVID-19, social capital, health, mental health, digital divide

## Abstract

**Background:**

During the COVID-19 pandemic, information and communication technology (ICT) became crucial for staying connected with loved ones and accessing health services. In this scenario, disparities in ICT use may have exacerbated other forms of inequality, especially among older adults who were less familiar with technology and more vulnerable to severe COVID-19 health consequences.

**Objective:**

This study investigated changes in ICT use, psychological and physical health, and social capital before and after the pandemic among the oldest old population (aged 80 years or older after the pandemic) and explored how internet use influenced these changes.

**Methods:**

We leveraged data from the InveCe.Ab study, a population-based longitudinal cohort of people born between 1935 and 1939 and living in Abbiategrasso, a municipality on the outskirts of Milan, Italy. Participants underwent multidimensional assessment at baseline (2010) and after 2, 4, 8, and 12 years. We restricted our analysis to cohort members who participated in the last wave (ie, 2022) and who did not have a diagnosis of dementia (n=391). We used linear mixed models to assess the impact of COVID-19 and time on changes in social capital, physical and psychological health, and ICT use in a discontinuity regression design while controlling for age, sex, education, and income satisfaction. Then, we assessed the influence of internet use and its interaction with COVID-19 on these changes.

**Results:**

COVID-19 had a significant impact on social relationships (β=–4.35, 95% CI 6.38 to –2.32; *P*<.001), cultural activities (β=–.55, 95% CI –0.75 to –0.35; *P*<.001), cognitive functioning (β=–1.00, 95% CI –1.28 to –0.72; *P*<.001), depressive symptoms (β=.42, 95% CI 0.10-0.74; *P*=.009), physical health (β=.07, 95% CI 0.04-0.10; *P*<.001), and ICT use (β=–.11, 95% CI –0.18 to –0.03; *P*=.008). Internet use predicts reduced depressive symptoms (β=–.56, 95% CI –1.07 to –0.06; *P*=.03) over time. The interaction between internet use and COVID-19 was significant for cultural activities (β=–.73, 95% CI –1.22 to –0.24; *P*=.003) and cognitive functioning (β=1.36, 95% CI 0.67-2.05; *P*<.001).

**Conclusions:**

The pandemic had adverse effects on older adults’ health and social capital. Contrary to expectations, even ICT use dropped significantly after the pandemic. Internet users maintained higher psychological health regardless of time and COVID-19 status. However, COVID-19 was associated with a steeper decline in cognitive functioning among internet nonusers. Policy makers may develop initiatives to encourage ICT adoption among older adults or strengthen their digital skills.

**Trial Registration:**

ClinicalTrials.gov NCT01345110; https://clinicaltrials.gov/study/NCT01345110

## Introduction

### Information and Communication Technology Use Among Older Adults in Italy

In the last few decades, the use of information and communication technology (ICT) has become pervasive and consistently more frequent at all ages [1,2]. The digitalization of public services has made it necessary for citizens to develop a certain degree of confidence in using digital devices [3]. In addition to population aging and contextual factors, this situation has contributed to an increase in the number of older adults who use ICT [4].

Indeed, the most recent Eurostat data shows that in 2023, the prevalence of internet use among the Italian population in the age group of 75 years or older has reached 25.7%, up from 13.1% in 2019. However, this strong increase in internet use among the oldest old segments of the population does not even remotely bridge the grey digital divide, that is, differences in technology adoption among the younger and oldest segments of the population. Indeed, internet use among individuals of all ages in Italy is 60 percentage points higher, reaching 87.7% in 2023 [5].

A growing number of studies have focused on different aspects of older individuals’ use of ICT, including social media [6], smartphones [7], and computers [8]. Concurrently, the concept of the “digital divide,” that is, the difference between those who have access to and use (effectively) digital media and those who do not [9], has become highly relevant in the literature on ICT use among older adults [10,11].

One key area of investigation is the relationship between digital inequalities, access to health services, and social capital formation and maintenance, which is often fueled by digital interactions. Given recent research [12] that has advocated for the adoption of a multidimensional perspective in the study of inequalities, this study focuses on the interrelation between digital inequalities and inequalities in health and social capital in the context of the COVID-19 pandemic using longitudinal data on the oldest old segments of the population (older than 80 years) living in the area of Abbiategrasso (Italy).

This study’s context is particularly interesting in the context of research on older adults’ habits and health, as well as the impact of COVID-19 on these dimensions. Indeed, Italy has one of the oldest populations in the world. Abbiategrasso is in Lombardy, a region that was among the first to be affected by the COVID-19 pandemic in the West, with dramatic consequences on the mortality rate, health, and social life of older adults.

### Social Capital and ICT Use Among Older Adults

One of the main motives behind ICT use is the desire to stay connected with others. As a matter of fact, several existing studies on the issue have focused on the effects of ICT use on social capital, as Warburton et al [13] highlighted in their review. In this respect, one key challenge in this literature is the lack of consensus on the operationalization of social capital [14]. A pioneering work by Putnam et al [15] defined social capital as a set of networks, rules, and trust that yields mutual benefits. An alternative interpretation is the one by Bourdieu [16] who viewed social capital as the sum of potential resources that people can use when they are members of a group. Alternatively, Coleman [17] interpreted social capital as the construct that facilitates collective action within a social structure and distinguishes social capital within and outside the family.

Notwithstanding the aforementioned different interpretations of social capital, its relationship with ICT use is bidirectional. On the one hand, the possibility of staying connected with others is one of the main motives for the use of ICT among the general population and in old age. On the other hand, building and maintaining social relationships, especially intergenerational relationships, is crucial to helping older adults improve their digital skills. However, the vast literature on the relationship between ICT use and social capital in old age (for a review, see [13]) has numerous limitations due to a lack of consensus in the operationalization of social capital, lack of identification of mechanisms that drive the formation and maintenance of social capital in old age, and limited focus on the oldest old people (those aged older than 80 years).

An additional level of difficulty is related to the role of ICT in the formation and maintenance of social capital among older adults. With the increasingly widespread use of digital media, the very concept of social capital may change [18] to include forms of socialization other than physical meetings and in-person participation in groups. Accordingly, research on this topic has considered the role of ICT use in these new forms of social capital [19]. The influence of ICT in stimulating face-to-face interactions (and other forms of social capital in its “traditional” sense, as presented above) has been widely investigated [20,21]. In addition, research has explored the effects of ICT use on dimensions of social capital, such as social participation, social exclusion, and loneliness [22]. Nonetheless, additional research is needed not only to validate and replicate prior research results but also to reflect on how, in the postpandemic context, continuously evolving digital technologies and new forms of digital interactions may foster social capital.

### Health and ICT Use Among Older Adults

The relationship between ICT use and health in older individuals seems complex. This is due not only to the multifaceted nature of health but also to the diverse ways ICT can be used in the context of health care, ranging from telehealth to the e-booking of medical appointments to web-based searches for health-related information.

Research on cognitive function shows that nondaily users of digital tools have overall lower cognitive performance than daily users [23], highlighting the beneficial effects of the use of digital tools on memory processes [24]. With respect to psychological health, research shows that people who use ICT experience fewer depressive symptoms and greater well-being [25-28].

The effects of ICT use on physical health in older adults have been studied less extensively. The literature mostly focuses on rehabilitation protocols using digital tools such as mobile apps. In this respect, the use of digital devices has proven to be effective in improving older people’s autonomy in daily activities [29] and recovery from falls [30], which are likely to occur in old age [31].

Since multimorbidity is a relatively frequent condition in old age [32], studying the impact of ICT use on the management of related symptoms is a particularly interesting endeavor. Research has shown that the use of digital technologies by older people with two or more long-term health conditions can help them face day-to-day issues related to symptom management and self-reported perceptions of health conditions [33]. Overall, it seems that the use of digital technologies has positive effects on managing symptoms of coexisting pathologies and their perceived severity in old age.

### The Impact of the COVID-19 Pandemic on Older Adults’ Social Capital, Health, and ICT Use

The COVID-19 pandemic had a remarkable impact on all the dimensions of interest in this research: social capital, health, and ICT use. First, social distancing measures limited older people’s ability to meet with family members and friends and hindered the possibility of participating in face-to-face volunteer work, associations, and activities within the local community [34].

Several activities have been moved to the digital sphere. Older people seem to have shifted traditional face-to-face relationships to nonphysical contact through video calls, social messaging, and social media [35]. In this respect, the pandemic seems to have accelerated the digitalization of society, and in fact, the importance of access to and the ability to proficiently use digital technologies in day-to-day activities from socializing to accessing health services. Indeed, recent research has documented a large amount of ICT adoption among the older people population during the pandemic. It seems that the shift from in-person to web-based activities during the most acute phase of the pandemic encouraged older adults to start or continue using digital technologies [36].

With respect to health, the COVID-19 pandemic clearly had important direct and indirect effects on the physical and mental health of older people. First, as widely documented, the severity of symptoms and the COVID-19–related mortality rate increase with age [37]. However, the pandemic may also have had a strong indirect impact on the health and well-being of older people [38,39]. In particular, the congestion of several nations’ health services due to the unprecedented demand for health care resulted in delays in diagnostic and therapeutic medical visits for health conditions other than those strictly related to COVID-19. This circumstance may have led to medical conditions that required attention to be overlooked.

Other studies have examined the role of ICT use in recovery from the SARS-CoV-2 infection. On this matter, existing protocols, which are regularly under evaluation [40], show that the use of digital devices to recover from COVID-19 is favored by patients [41] and allows more personalized treatment plans [42]. While limited evidence exists to date, it is reasonable to anticipate a growing body of research in this area in the future.

### Research Questions and Hypotheses

The evidence discussed above shows a multifaceted state of the art. While the number of studies investigating this topic has recently increased, existing research still has numerous potential shortcomings that this study aims to address.

First, limited research has been conducted on the effects of ICT use on health and social capital in adults aged 80 years and older, a rapidly growing portion of the population with unique health and social care needs. However, this topic has rarely been studied by researchers. We believe that this methodological choice might lead to an incomplete understanding of the issue. This research aims to fill this gap by studying the effects of ICT use with data from a sample of people around and older than 80 years.

Second, most existing studies were cross-sectional [43,44]. This type of research design is suitable for studying the associations between different dimensions, but it is not optimal for drawing conclusions on the direction of these associations. One limitation that affects the literature on the relationship between ICT use and health is a lack of assessment of the direction of the association between these two dimensions. While some of the aforementioned studies seem to suggest that ICT use drives better health outcomes, good health could be seen as a prerequisite (and a driver) for the acquisition of digital skills and digital technology adoption. Accordingly, longitudinal studies provide a favorable environment to establish the order of events (whether ICT adoption is associated with subsequent health outcomes), aiding in the assessment of causation [45].

Furthermore, analyzing prospective longitudinal cohort data is appropriate for capturing changes over time, which is crucial for studying a rapidly growing and dynamic phenomenon such as ICT use in older individuals.

Third, many studies on this topic have been conducted using qualitative research designs [46,47]. The advantages of these designs include a deeper investigation of subjective experiences and the possibility of investigating complex concepts without being bound to a predefined set of possible responses. However, qualitative studies lack standardized measurement, which is crucial for tracking evolution over time and assessing the magnitude of effects.

Furthermore, most existing studies have focused on only some of the dimensions considered in this study, namely, the effects of ICT use on cognitive functioning [48] and social relationships [43]. However, to our knowledge, no study to date has explored the evolution of ICT use, cognitive functions, and social relationships concurrently over time while adopting a multidimensional and multidisciplinary approach.

Considering the above, this research’s objective is 2-fold. First, this study assesses the effects of the COVID-19 pandemic on changes in the ICT use, social capital, and physical and psychological health of the oldest old population. Second, this study aims to investigate whether ICT use plays a role in mitigating the impact of the COVID-19 pandemic on the aforementioned dimensions.

Specifically, two research questions (RQs) guided the investigation presented in this work.

RQ1: Is there any change in ICT use, psychological and physical health, and social capital before and after the pandemic among the oldest old population?

RQ2: Do higher levels of ICT use have an impact on psychological and physical health and social capital over time in the context of the COVID-19 pandemic?

In light of current knowledge on this topic, we hypothesized that during the COVID-19 pandemic, health and social capital deteriorated while ICT increased. We also hypothesized that prepandemic ICT use is positively associated with social capital and health as it facilitates interpersonal communication, promotes social gatherings, and facilitates recovery from health issues.

## Methods

### Study Context

The data used in this research were collected as part of the study InveCe.Ab [49], a population-based cohort study conducted in Abbiategrasso, a municipality on the outskirts of the Metropolitan City of Milan (Italy). This study aimed to analyze the effects of aging on cerebral functions and the biological, social, clinical, and neuropsychological factors related to the onset of dementia. The data were collected at baseline (2010) and at follow-ups (2012, 2014, 2018, and 2022). The target population included older adults born between 1935 and 1939 who resided in Abbiategrasso on the prevalence day (November 1, 2009). Data in each wave were collected through a multidimensional assessment that included a social and lifestyle questionnaire, a medical visit, a neuropsychological evaluation, and the collection of anthropometric measures and blood samples.

### Participants

**Figure 1:**
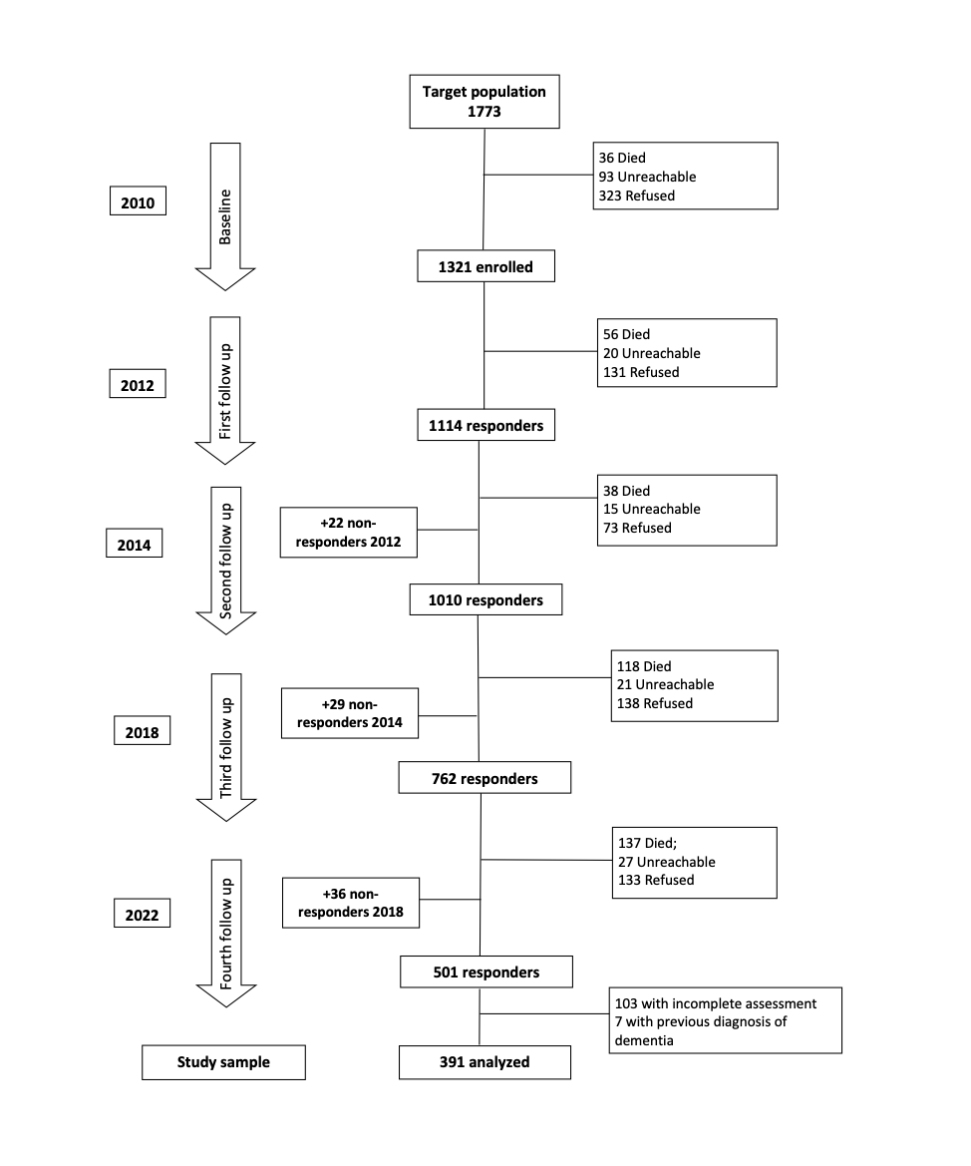
Flowchart of the InveCe.Ab study and participants selection for the present investigation. At baseline, we enrolled 1,321 individuals from the target population (recruitment rate: 80.35%). At each follow-up, all alive InveCe.Ab participants still residing in Abbiategrasso were recontacted. Reasons for non-participation at each wave are detailed on the right side of the flow chart, while, on the left side, we reported the number of participants enrolled among those who did not participate in the previous follow-up.

The baseline sample of the InveCe.Ab study included 1321 people. Figure 1 shows the flowchart of study participation throughout the observation period (12 years, from 2010 to 2022). In this work, we analyzed data from a subsample composed of people who participated in the 2022 wave (after the COVID-19 outbreak), who were not previously diagnosed with any form of dementia, and who completed the full version of the questionnaire on social and lifestyle variables in the postpandemic wave in 2022 (n=391). We excluded sample members diagnosed with dementia from the analysis because we assumed that cognitive impairment could lead to measurement errors in self-reported health and social relationships, as well as endogeneity. Moreover, we excluded data collected through a shortened version of the social and lifestyle questionnaire since it did not include some of the variables considered in this study. We compared the main sociodemographic features at baseline between the study sample (n=391) and the InveCe.Ab participants who were not included, excluding 156 cases with incident dementia (from 2010 to 2018) and 8 with incomplete baseline assessment (n=766). They were comparable for sex (study sample=57% female, n=223; unselected=53%, n=404 female; *P*=.17), while the study sample was slightly younger (study sample mean 72.0, SD 1.3; unselected mean 72.2, SD 1.3; *P*=.02) and highly educated (study sample mean 7.4, SD 3.3; unselected mean 6.6, SD 3.3; *P*<.001).

### Measures

In the following, we describe the measures used in the analysis.

#### ICT Use

ICT use was measured with the following three questions: “Do you use a mobile phone?” “Do you use a computer?” and “Do you use the internet?” The answer options were “yes,” “no,” and “I don’t know.” A derived variable was computed as the sum of the 3 questions.

#### Social Capital

To capture the multidimensional nature of social capital as much as possible, we computed two variables, namely, “Social relationships” and “Cultural activities.” The former was the product of the monthly frequency of in-person contact with family, friends, and colleagues, ranging from 0 (there are none or not living together) to 2 (we meet frequently [four or more times a month]), and self-reported satisfaction with these contacts, ranging from 0 (not at all satisfied) to 4 (very satisfied).

The “Cultural activities” variable was computed as the sum of cultural activities (in the last year) that entailed contact with other people. These included going to the cinema or the theatre, attending courses, volunteering, participating in associations or groups, and playing cards. Responses ranged from 0 (no involvement) to 2 (frequent involvement).

#### Physical Health

This dimension was quantified with the Cumulative Illness Rating Scale (CIRS) [50], a 14-category measure of clinical and functional acuteness. The first 13 elements of the CIRS scale were averaged to form the CIRS severity index.

#### Psychological Health

The assessment of psychological health comprised 2 dimensions: cognitive functioning and mood. The former was explored with the administration of the Mini-Mental State Examination, which is a screening test used to detect cognitive impairment [51]. The latter was assessed with the Geriatric Depression Scale, a self-reported measure of depressive symptoms in old age [52].

#### Time

The passing of time was measured with a variable that could take five values (2010, 2012, 2014, 2018, and 2022) corresponding to the years the InveCe.Ab waves took place.

#### COVID-19

The presence or absence of the COVID-19 pandemic in a specific wave was operationalized with a dichotomous variable that took a value of 1 in 2022 and 0 in all other years.

#### Sociodemographic Variables

Age at baseline, sex, education, and the perception of the adequacy of one’s income were considered relevant confounding factors due to their established association with ICT adoption and use.

### Statistical Analysis

#### Power

To determine the minimum sample size required for the analyses, we conducted a power analysis with G*Power (version 3.1.9.7) [53]. Under the linear multiple regression-random model group, we set the effect size at ρ2=0.05, the significance criterion at α=0.05, and the power at 0.80. Our sample size was sufficiently large to conduct the analyses, as it exceeded 309, which is the sample size required for conducting this study, based on the aforementioned calculations.

#### Data Analysis

Inferential analysis was conducted using R [54] and RStudio [55].

To investigate the effects of the pandemic on changes in the dimensions of interest (RQ1), we ran a series of linear mixed models (LMMs) with social capital, physical and psychological health, and ICT use as the outcomes and time and COVID-19 as the predictors in a discontinuity regression design [56]. Age, sex, education, and the perception of the adequacy of one’s income were included as controls. Since we leveraged longitudinal data with repeated measures over the waves, we computed these models with random intercepts for the participants. To further assess the impact of the COVID-19 pandemic in a discontinuity regression framework, we compared the predicted values (PVs) of the postpandemic wave (2022) based on the prespecified models considering the first four waves (2010-2018) and compared them with the observed values (OVs) measured in 2022 with 2-tailed paired-sample *t* tests.

To address RQ2, we used the same approach but included: internet use and the interactions between internet use and time, and between internet use and COVID-19, as predictors, and time, COVID-19, age, sex, education, and the perception of the adequacy of one’s income as covariates. The COVID-19 variable was conceived as a dichotomous variable that took a value of 1 in 2022 and 0 in all other years, while time was calculated as years from baseline. We adopted internet use as a predictor (rather than the abovementioned ICT use-derived variable) because web-based activities are likely to have a greater impact on the variables of interest than other forms of ICT use. Thus, this model included a time-varying predictor (internet use) and time-varying covariates.

### Ethical Considerations

The study procedures were in accordance with the principles outlined in the Declaration of Helsinki of 1964 and the subsequent amendments. The InveCe.Ab study protocol was approved by the Ethics Committee of the University of Pavia on October 6, 2009 (Committee report 3/2009).

## Results

### Sample Features

Descriptive statistics are reported in Table 1. On average, the participants were 72 (SD 1.3) years old when the InveCe.Ab study started (2010) and had an average education level of 7.4 (SD 3.3) years. The majority of the sample was composed of female participants (n=223, 57%). Most people perceived their income as adequate or just enough. In the prepandemic wave conducted in 2018, approximately 7% (n=30) of the participants reported that they did not use any ICT devices, while approximately 22% (n=87) were internet users.

**Table 1:** Descriptive statistics of the sample at baseline (2010).

Variables			Study sample (N=391)
**Sociodemographic characteristics**			
	Age at baseline (years), mean (SD)	72.0 (1.3)	
	Sex (female), n (%)	223 (57)	
	Education, mean (SD)	7.4 (3.3)	
**Perceived adequacy of one’s income, n (%)**			
	Not adequate	30 (7)	
	Just enough	171 (43.7)	
	Adequate	190 (48.6)	
**Variables of interest, mean (SD)**			
	Social relationships	51.5 (16.8)	
	Cultural activities	6.6 (1.9)	
	Physical health (CIRS^a^ severity index)	1.5 (0.3)	
	Cognitive functioning (MMSE^b^)	27.8 (2.3)	
	Depressive symptoms (GDS^c^)	2.3 (2.6)	
Internet use, n (%)			51 (13)
Computer use, n (%)			72 (18)
Mobile phone use, n (%)			350 (89.5)

^a^CIRS: Cumulative Illness Rating Scale.

^b^MMSE: Mini-Mental State Examination.

^c^GDS: Geriatric Depression Scale.

Over the years, our sample has shown a steady increase in the percentage of internet users, rising from 13% (n=51) in 2010 to 16% (n=64) in 2012, 17% (n=67) in 2014, and eventually reaching 27% (n=106) by 2022. Meanwhile, the proportion of sample members who do not use ICTs has remained relatively stable, fluctuating slightly around 9% throughout the period, with specific figures of 8% (n=34) in 2010, 9% (n=38) in both 2012 and 2014 (n=36), and 10% (n=42) in 2022.

### The Impact of the COVID-19 Pandemic

Table 2 shows the results of the LMM models used to evaluate the effect of COVID-19 on the longitudinal changes in the measures of interest.

COVID-19 appears to have reduced social relationships (β=–4.35; conditional *R*^2^=0.58), cultural activities (β=–.55; conditional *R*^2^=0.67), cognitive functioning (β=–1.00; conditional *R*^2^=0.57), and ICT use (β=–.11; conditional *R*^2^=0.77), and increased depressive symptoms (β=.42; conditional *R*^2^=0.58) and multimorbidity (β=.07; conditional *R*^2^=0.57). These effects were independent of time, sex, age, education, and perception of the adequacy of one’s income.

These results were further supported by 2-tailed paired-sample *t* tests that tested the differences between the mean PVs on the basis of waves 1 through 4 and the mean OVs in wave 5. Such *t* tests showed that the participants’ social relationships (mean difference=–4.37; *P*<.001; PV=48.66; OV=44.39), cultural activities (mean difference=–0.56; *P*<.001; PV=6.36; OV=5.80), cognitive functioning (mean difference=–1.01; *P*<.001; PV=27.49; OV=26.51), and ICT use (mean difference=–0.10; *P*<.001; PV=1.42; OV=1.32) after the spread of the COVID-19 pandemic were significantly lower than expected. Concurrently, multimorbidity (mean difference=0.07; *P*<.001; PV=1.62; OV=1.68) and depression (mean difference=0.43; *P*<.001; PV=2.97; OV=3.42) were significantly greater than expected.

**Table 2:** Linear mixed models estimating the effect of COVID-19 on social relationships, cultural activities, cognitive functioning, depressive symptoms, physical health, and ICT^a^ use.

	Estimates (SE)	95% CI^b^	*P* value	ICC^c^
Social relationships	–4.35 (1.04)	–6.38 to –2.32	<.001^d^	0.53
Cultural activities	–0.56 (0.10)	–0.76 to –0.37	<.001^d^	0.61
Cognitive functioning	–1.00 (0.14)	–1.28 to –0.72	<.001^d^	0.45
Depressive symptoms	0.42 (0.16)	0.10 to 0.74	.009^d^	0.50
Physical Health	0.07 (0.02)	0.04 to 0.10	<.001^d^	0.54
ICT use	–0.11 (0.04)	–0.18 to –0.03	.008^d^	0.69

^a^ICT: information and communication technology.

^b^CIs were computed using the Wald method. The control variables are time, sex, age, education, and income adequacy.

^c^ICC: IntraClass Correlation coefficient.

^d^*P* values <.05.

### The Role of Internet Use

**Figure 2:**
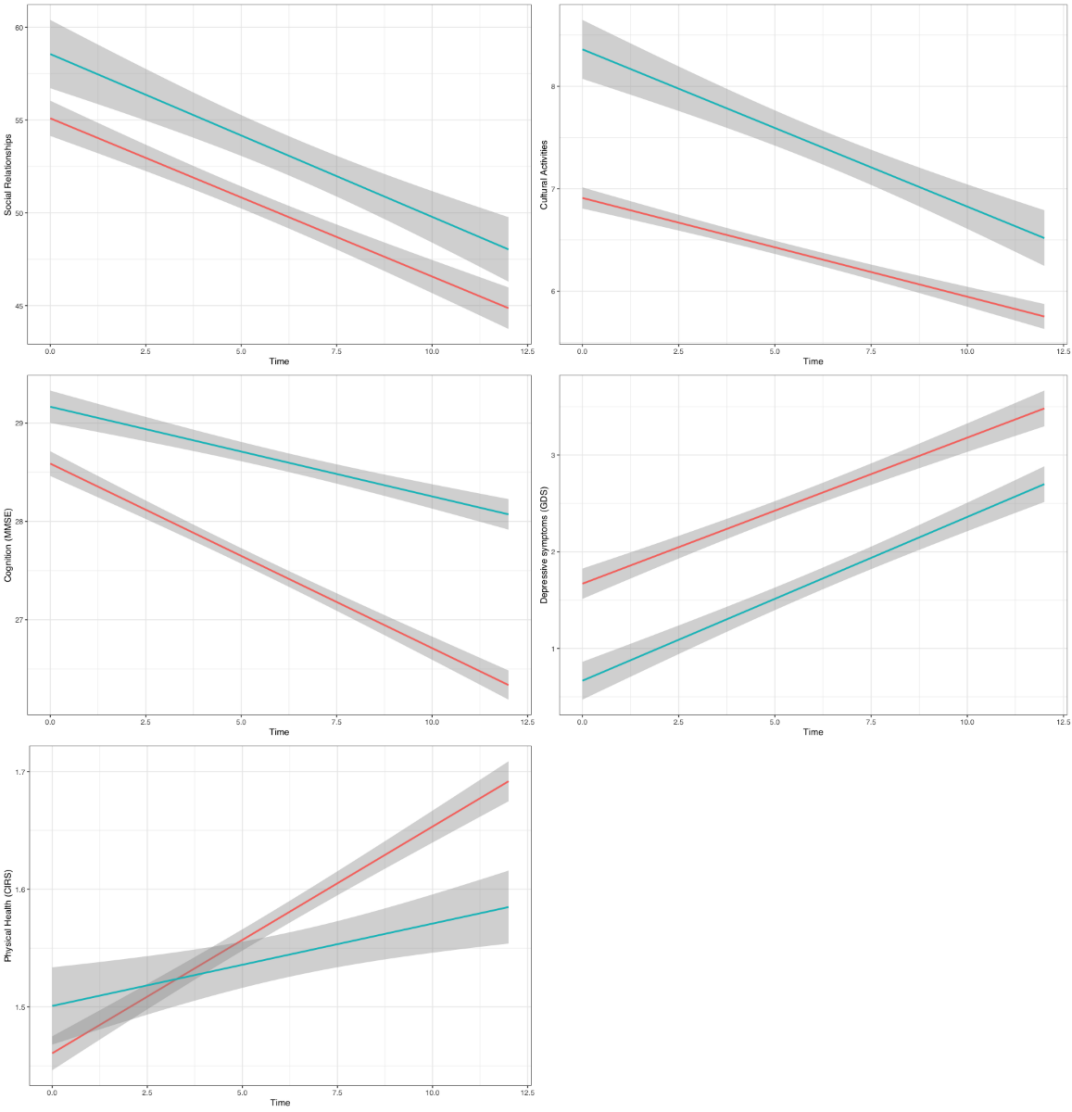
Visual representation of the changes in the measures of interest according to internet used status based on the linear mixed models performed (research question 2). The blue line represents internet users, and the red line represents nonusers. CIRS: Cumulative Illness Rating Scale; GDS: Geriatric Depression Scale; MMSE: Mini-Mental State Examination.

Table 3 shows the results of the LMM models assessing the effect of internet use on the longitudinal changes in the measures of interest. Figure 2 shows the changes in the measures of interest across the assessment wave stratified by internet use.

We found that internet use had a significant effect on depressive symptoms (β=–.56; conditional *R*^2^=0.58) while only a marginally significant effect (*P*<.10) was found for social relationships (β=2.91; conditional *R*^2^=0.58) and cultural activities (β=0.28; conditional *R*^2^=0.68). The interaction term between internet use and time was not significant, indicating that the effects of internet use on the measures of interest were stable across waves. However, the interaction between internet use and COVID-19 was significant for cultural activities (β=–0.73; conditional *R*^2^=0.68) and cognitive functioning (β=1.36; conditional *R*^2^=0.58), thus indicating that the effect of internet use on the measures of interest was modified by the pandemic, resulting in a steeper decline for internet users in cultural activities and a steeper decline in cognitive functioning for internet nonusers. A trend toward the significance of the interaction term was further found for physical health (β=–.07; conditional *R*^2^=0.58). These effects were independent of time, COVID-19 status, sex, age, education, and the perception of the adequacy of one’s income.

**Table 3:** Linear mixed models estimating the effect of internet use on social relationships, cultural activities, cognitive functioning, depressive symptoms, and physical health during the COVID-19 pandemic.

		Social relationships	Cultural activities	Cognitive functioning	Depressive symptoms	Physical health
**Internet use**						
	Estimates (SE)	2.91 (1.67)	0.28 (0.17)	0.16 (0.22)	–0.56 (0.26)	0.03 (0.03)
	95% CI^a^	–0.37 to 6.18	–0.05 to –0.60	–0.42 to 0.45	–1.07 to –0.06	–0.02 to 0.09
	*P* value	.08	.09	.94	.03^b^	.19
**Interaction between internet use and time**						
	Estimates (SE)	0.10 (0.26)	–0.00 (0.03)	–0.00 (0.04)	–0.01 (0.04)	–0.01 (0.00)
	95% CI	–0.41 to 0.62	–0.05 to 0.05	–0.07 to 0.07	–0.09 to 0.07	–0.01 to 0.00
	*P* value	.69	.95	.99	.74	.18
**Interaction between internet use and COVID-19**						
	Estimates (SE)	–0.22 (2.58)	–0.73 (0.25)	1.36 (0.35)	0.30 (0.40)	–0.07 (0.04)
	95% CI	–5.27 to 4.83	–1.22 to –0.24	0.67 to 2.05	–0.49 to 1.09	–0.15 to 0.01
	*P* value	.93	.003^b^	<.001^b^	.46	.08
	ICC^c^	0.53	0.62	0.45	0.50	0.55

^a^CIs were computed using the Wald method. The control variables are time, sex, age, education, and income adequacy.

^b^*P* values <0.05.

^c^ICC: intraclass correlation coefficients.

## Discussion

### Principal Results

This study explored the impact of the COVID-19 pandemic on social capital, health, and ICT use among older adults (RQ1) and investigated the effects of internet use on older adults’ social capital and health throughout time and in the context of the COVID-19 pandemic (RQ2).

With regard to RQ1, our results largely confirmed our hypotheses. In particular, the COVID-19 pandemic predicted reduced social capital in terms of social relationships and cultural activities that entail contact with other people. In addition, the results indicated that the pandemic was predictive of worsened physical condition. Consistently, we found that the pandemic predicted lower levels of psychological health, which was assessed through the Mini-Mental State Examination and Geriatric Depression Scale to capture different aspects of this dimension, namely, cognitive functioning and depression. With respect to ICT use, we expected that the COVID-19 pandemic would predict heightened ICT use. This hypothesis has roots in the body of knowledge showing that the pandemic yielded higher levels of use of digital technologies among older adults to stay connected with family and friends as a result of restrictions on in-person contact imposed by authorities. However, this hypothesis was not confirmed by the study results. In contrast, we found that the pandemic predicted a lower level of ICT use.

With respect to RQ2, the results partially confirmed the hypotheses. More specifically, internet use was found to be positively associated with older adults’ psychological health. Our results confirmed this hypothesis. Internet use was inversely related to depressive symptoms over time. Additionally, we found that COVID-19 modified the relationship between internet use and cognitive functioning, with nonusers showing a steeper decline in cognitive functioning during the pandemic.

We also expected internet use to be predictive of higher social capital levels. However, internet use did not predict the number of cultural activities that involve contact with other people, such as attending courses and participating in associations. This may be because training and associateship still follow channels other than digital channels. Interestingly, the relationship between internet use and cultural activities was modified by the pandemic and showed a greater reduction in cultural activities related to internet use. During the COVID-19 pandemic, cultural activities that entailed contact with other people were significantly limited and not recommended, especially for older adults. We, therefore, speculate that the impact was greater for those who were habitually more involved in these kinds of activities. Although the participants’ sociodemographic characteristics were accounted for in our models, we cannot rule out the possibility that internet users might have a sociodemographic profile that entails greater involvement in cultural activities that imply contact with other people. This is confirmed by internet users’ higher levels in this dimension at the baseline, as Figure 2 suggests.

Moreover, internet use is not significantly correlated (at standard significance levels, ie, *P*<.05) with the level of social relationships, which is the other variable considered in this study for the assessment of social capital. This lack of association could be attributed to the fact that our measure of social relationships measures the frequency of in-person interactions with family, friends, and colleagues. Instead, internet use may have primarily facilitated increased communication through digital channels rather than face-to-face contact.

It should be noted, however, that the cut-off for statistical significance may be relaxed in our analysis in light of the relatively small sample size. As such, we consider marginally statistically significant the association between internet use and social relationship (*P*=.08) and cultural activities dimensions (*P*=.09).

Finally, internet use did not affect physical health. A possible explanation for this result is that the oldest-old segments of the population might use the internet mainly for communicating and may lack the skills required for digital access to health care services.

### Comparisons With Previous Work

With regard to RQ1, our results align with the findings of previous studies on the negative effects of the COVID-19 pandemic on social activities [57,58]. In relation to physical health, our results are in line with those of other studies showing that older adults are more vulnerable than other age groups to COVID-19 symptoms [59]. This outcome is consistent with existing evidence regarding older people’s health susceptibility to unfavorable consequences of viral infections [60]. Regarding the adverse impact of the pandemic on psychological health, our results reflect existing knowledge on the topic that highlights the adverse impact of the pandemic on older adults’ health, such as lower cognitive functioning [61] and higher levels of depression [62]. With respect to the negative effect on ICT use, this finding might be explained by the return to in-person contact after the pandemic (in 2022, when our postpandemic data were collected) and the resulting lower levels of digital technology use by older adults. Another aspect should be considered when interpreting these results: given the unexpected and extraordinary nature of the pandemic, older people may have faced these challenging times with the skills and tools that they had already mastered, which did not necessarily include ICT devices. Indeed, in our sample, only 13% (n=51) of older adults used the internet at baseline, and 22% (n=87) used it in the last wave before the pandemic (ie, 2018). Rather than increasing ICT take-up, this age group may have turned to alternative sources of support. Such interpretation may be confirmed by the percentage of people who reported no use of ICT at all (around 10%, n=42) versus the percentage of ICT nonusers in 2018 (around 7%, n=30). Additionally, a possible decline in participants’ physical and cognitive conditions may have led to a lower level of ICT use.

Moving to RQ2, the lack of statistically significant predictive effect of internet use on greater social capital is consistent with the groundbreaking study conducted by Kraut et al [63], showing that greater internet use in older age was associated with lower levels of communication with relatives in the household and reduction in the social circle size. This result was partially corroborated by the study by Zhao [64], which showed that people who use the internet for nonsocial purposes do not differ from internet nonusers when it comes to network size.

In relation to the effects of internet use on psychological health, our results are consistent with existing studies that show an overall positive effect of ICT use on older adults’ psychological health [65]. The effects of internet use on cognitive functioning may have different interpretations. Several studies have shown that the use of technology fosters cognitive function since it requires several cognitive skills. For instance, Choi and Hart [66] reported that greater ICT use is associated with better performance in tasks related to episodic memory and executive functions. Similarly, Yeung et al [67] demonstrated that higher levels of ICT use are associated with increased working memory and visuospatial ability. On the other hand, we cannot exclude reverse causality, with increased cognitive decline due to COVID-19 explaining a reduction in internet use.

The lack of effects of internet use on physical health is consistent with prior research that highlighted the sense of helplessness and insecurity of older adults in accessing digitally led health care [68]. Such concerns are greater in rural areas, where older adults show even lower levels of digital literacy and technology acceptance associated with health care [69]; hence, these findings may also apply to the suburban context of Abbiategrasso. Further research should explore different types of internet use among the oldest old people.

In summary, our results integrated the current understanding of the topic and added new layers of knowledge. The longitudinal research design allowed us to explore the medium- to long-term effects of the dimensions considered herein, namely, older adults’ ICT use, social capital, and health. In addition, we studied the impact of the COVID-19 pandemic on these dimensions. Overall, the pandemic seemed to have a widespread negative impact on all of these aspects. Nonetheless, internet use seemed to predict better psychological health levels over time. Thus, internet use is a protective factor as people age, independent of sociodemographic characteristics and the effect of COVID-19. We also found that COVID-19 modulated the relationships between internet use and several cultural activities and cognitive functioning, although in opposite directions.

This work has several strengths. First, to our knowledge, this is the first work that analyses ICT use, social capital, and health in the oldest old population (aged older than 80 years) and is based on probability-based longitudinal data and multidimensional assessments collected over a 12-year period. In particular, medical and neuropsychological evaluations were combined with a self-reported appraisal of social activities and lifestyles to provide detailed information on each dimension.

Second, participants from a limited geographical area were included in this study, which limited the potential biases that might arise when the sample comprises individuals from different communities. Moreover, the fact that the data collection waves were conducted before and after the most acute phase of the COVID-19 pandemic allowed us to compare the impact of this event on the dimensions of interest.

Finally, by adopting a longitudinal design, we could observe ICT use at baseline and outcomes of interest at later time points. This allowed us to overcome one key methodological limitation of the literature, which has adopted cross-sectional designs and failed to identify the direction of associations between the use of technologies, health, and social capital.

### Limitations

This research has several limitations. First, since this was an observational study and did not entail the experimental manipulation of the variables of interest, the exploration of the direction of causal inferences has a certain degree of ambiguity. Additionally, ICT was measured as a dichotomous variable that assessed only use or nonuse. A more refined approach would capture nuances in the frequency of ICT use. Furthermore, the longitudinal data were limited to one wave after the most acute phase of the pandemic. Future research may replicate this analysis should additional survey waves become available. Finally, the generalizability of our results to populations with different sociodemographic characteristics is limited. Future studies may address this issue by conducting similar research in different contexts and among populations with more heterogeneous characteristics.

### Conclusions

This study assessed the impact of the pandemic on older adults’ health and social capital and explored the role of inequalities in ICT access by leveraging a population-based sample of older adults followed by a multidimensional assessment over a 12-year period.

We believe our results will be of interest not only to the scientific community but also to policy makers by assisting them in assessing the importance of fostering ICT use in old age and drafting policy measures accordingly. First, the pandemic significantly affected multiple dimensions of older adults’ lives. Measures aimed at aiding older people are particularly necessary in areas most significantly affected by the pandemic. As internet use seems to have beneficial effects on psychological health in older adults, policy makers may develop initiatives that encourage the adoption of digital devices among older adults or strengthen their digital skills. Such initiatives may include but are not limited to, intergenerational educational programs through which young adults act as “cyber tutors” for older people [70,71] and courses to improve digital literacy in old age [72].

This work seeks to enrich the current body of knowledge on the effects of ICT use on older adults’ social capital and health. This study provides direct evidence on the longitudinal trajectory of these relevant dimensions in a population-based sample of older adults residing in a specific context during the COVID-19 pandemic. Future research may address the limitations of this study and further develop a thorough understanding of these rapidly changing topics.

## Abbreviations

CIRS: Cumulative Illness Rating Scale
ICT: information and communications technology
LMM: linear mixed model
OV: observed value
PV: predicted value
RQ: research question

## Data Availability

The datasets generated and analyzed during this study are available from the corresponding author upon reasonable request.
